# Energy production pathways of female soccer players during championships: a metabolomics approach

**DOI:** 10.1590/1414-431X2025e14589

**Published:** 2026-02-16

**Authors:** M.B.A. Nascimento, M.M.S. Gouveia, M.P.P. Santos, E.R. da Rocha-Junior, A.C. Crispim, E.S. Bento, T.M. Aquino, F.A.B. Sousa, G.G. de Araujo, T. Ataide-Silva

**Affiliations:** 1 Programa de Pós-Graduação em Nutrição, Faculdade de Nutrição, Universidade Federal de Alagoas, Maceió, AL, Brasil; 2 Laboratório de Ciências do Esporte Aplicadas, Instituto de Educação Física e Esportes, Universidade Federal de Alagoas, Maceió, AL, Brasil; 3 Núcleo de Análises e Pesquisa em Ressonância Magnética Nuclear (NAPRMN), Instituto de Química e Biotecnologia, Universidade Federal de Alagoas, Maceió, AL, Brasil

**Keywords:** Sportomics, Women, Energy metabolism, Nuclear magnetic resonance

## Abstract

Athletes mobilize both aerobic and anaerobic systems to produce energy during soccer matches, and it drastically modifies the concentration of metabolites related to muscle damage and energy metabolism. The fatigue-associated mechanism seems to encompass tricarboxylic acid cycle (TCA) disturbances, with a greater contribution of lipid-pathway metabolites in women during soccer matches. However, to the best of our knowledge, the effects of matches played during two championships on the metabolites and pathways associated with energy production in female players have not yet been described. Metabolomic analysis in sports context can better characterize the main metabolites related to energy production and the metabolic pathways. The aim of this study was to describe the variation in metabolites over the course of two championships in female players' urine, highlighting the occurrence of energy production-associated metabolites and the metabolic pathways they may arise from. Urine samples were collected before and after six matches of two championships. Nuclear magnetic resonance metabolomic approach was used for this purpose. Citrate, succinate, 1-methylnicotinamide, alpha-hydroxyisobutyrate, malonic and glycolic acids, phosphocreatine, and lactate were the compounds originated from energy generation processes with high scores in variable importance prediction (VIP), impacting on partial least squares discriminant analysis (PLS-DA) groupings of players. Phenylalanine biosynthesis, tyrosine and tryptophan biosynthesis, and the TCA cycle were mobilized before the matches, and the metabolism of taurine and hypotaurine were modulated across both moments.

## Introduction

Soccer is a team sport that requires athletes to oscillate at different physical intensity levels during the match, mobilizing both the aerobic and anaerobic systems to produce energy. This drastically changes metabolite concentrations associated with substances' catabolism and anabolism processes, muscle damage, and energy production (1-3). Although the application of metabolomics to soccer is recent (4), a few studies have already used metabolomics (1,5,6) to observe metabolic changes during soccer, since this approach overlaps traditional chemical analyses and allows assessing metabolic fluctuations in the body from an integrated point of view (5). Metabolomics involves the quantitative analysis of the metabolome, allowing researchers to assess physiological changes in the human body (1,7).

Metabolomics studies involving soccer players have enabled the description of adaptation to training, the likelihood of developing muscle injury conditions, muscle fatigue related mechanisms, and energy substrates (1,6,8-15). In a previous study on the manifestation of metabolites associated with energy production after a soccer match in young male athletes, the changes found seem to involve glycolytic and lipid metabolites (15). In another analysis, the authors carried out a metabolic pathway enrichment analysis (MetPA) and observed that bioenergetic metabolic pathways had an impact on soccer players, suggesting that the mechanism associated with fatigue is linked to disturbances in the tricarboxylic acid cycle (TCA) (1).

Based on the evidence of sex-specific differences in metabolism (16), it is possible to speculate that there is a greater contribution of metabolites from the lipid pathway in women than in men during a soccer match (14,16). However, as far as we know, no pre- or post-game changes in metabolites and energy pathways during championships in female soccer players have been described in the literature to date. Although disparities in phenotypes can significantly influence basal concentrations between external training loads in soccer (14), some studies have described metabolic variations in female footballers (12-14). Although these studies present a longitudinal analysis (14) or outline pre-match and post-match considerations (12,13), the metabolic changes observed in female footballers and the pathways mobilized by these compounds have not yet been investigated over the course of a championship. Considering that contextual elements such as the outcome of the match, the location of the match, the type of competition, and the period of the season can have an impact on the players' effort during a soccer match (17), it is necessary to characterize the changes in the metabolic profile considering a large number of matches.

Assessing female players' urine metabolites can contribute to improve knowledge about the required energy pathways and to plan more assertive strategies and prescriptions for training and diets of female soccer players. The current study hypothesis was that glucose and lipid pathway intermediates are among the higher variable importance prediction (VIP) scores in the metabolites identified in urine samples and with high impact scores in MetPA, through the changes in female soccer players during several matches of a championship. The aim of the present study was to describe the variation of metabolites in players' urine before and after matches of a championship, with focus on energy production-associated metabolites and their metabolic pathways.

## Material and Methods

### Participants

Non-probabilistic convenience sampling was adopted. Nineteen local professional female soccer players in the age group 19-32 years were invited to join the study. The athletes were informed about all the procedures to be conducted during the research, as well as the possible risks and benefits, and then signed the informed consent form. This study was part of a larger project entitled Analysis and Improvement of Athlete Performance approved by the Research Ethics Committee (CEP) of the Federal University of Alagoas (UFAL) under the protocol number CAAE: 29269020.8.0000.5013 and Opinion: 4297907, in compliance with ethical precepts in the Declaration of Helsinki.

### Experimental protocol

Anthropometric data (mass and height) were collected in a single visit during the pre-season using an anthropometric scale and a stadiometer (Adult Mechanical Scale 180 kg, Welmy^®^, Brazil), and skinfolds (triceps, supra iliac, and medial thigh) were measured with a skinfold caliper (lange Skinfold caliper, Cambridge Scientific Industries^®^, USA). Body fat percentage was calculated with the sum of skinfolds using the equation by Jackson et al. (18). Twenty-five milliliters of urine was collected before and after 6 matches, with an average interval of 10 days between matches (minimum: 1 day; maximum: 21 days), along two championships. The players participated in two simultaneous championships (state and national tournaments), and data were collected in 3 matches in each one of them.

### Metabolomic analysis

An aliquot of 1.5 mL of the samples was transferred to Eppendorf tubes and centrifuged at 19,000 *g* and 4°C for 15 min in a MIKRO 220R centrifuge (Hettich^®^, Germany). After centrifugation, 300 µL of the supernatant from each sample was transferred to a 5-mm NMR tube and 300 µL of 1 mM phosphate buffer solution (D_2_ O, pH=7.4, TSP=1 mM) was added. NMR spectra were acquired in a Bruker Avancer spectrometer (Bruker^®^, Germany*)* equipped with a superconducting magnet operating at 600 MHz and a 5-mm PABBO broadband probe, at 300 K, for hydrogen analysis by using the noesygppr1d pulse sequence to suppress the water signal by pre-saturation. The following parameters were adopted: 128 scans, 4 s between scans (D1), 64,000 spectrum points, 20 parts per million (ppm) window width, and 5.11-s acquisition time. Water signal appeared and irradiated (O1P) at 4.69 pmm.

The spectra were processed in TopSpin^®^ software, version 3.6.5, and the observed metabolites were identified at the Human Metabolome Database (HMDB) platform (www.hmdb.ca) and in Chenomxprofiler^®^ software (version 9.05). All spectra pre-processing for data-matrix acquisition was carried out in R software (version 4.2.2), using the PepsNMR package (version 3.17). All spectra were overlaid and aligned, and metabolites were relatively quantified in the PepsNMR package (version 3.17) to generate a table in .xls format with the samples in the rows and identified metabolites in the columns.

### Statistical analysis

Central tendency measurements and anthropometric analysis dispersion were carried out in Jamovi^®^ software (version 2.2.5). Partial least squares discriminant analysis (PLS-DA) was carried out in the Metaboanalyst^®^ website (version 5.0) using sum normalization, Pareto scaling, and logarithmic transformation. Initially, differential metabolites were found through Kruskal-Wallis test with the P-value corrected to the false discovery rate (FDR). Game was the grouping variable and relative metabolite concentrations were the dependent variables. Subsequently, MetPA was carried out in Metaboanalyst^®^ (version 5.0) by taking into consideration impact scores >0.02, -log(*p*) ≥1, and P value <0.05 to detect the affected pathways. The Kyoto Encyclopedia of Genes and Genomes (KEGG) library was used for MetPA.

## Results

Players who did not provide complete metabolic data were excluded from the sample. Thus, only 14 of the 19 players on the team completed the data collection and were included in the analysis. The athletes' anthropometric profiles are described in Table 1.

**Table 1:** Anthropometric profile of fourteen professional female soccer players.

Variables	Mean	Median	Standard deviation	Minimum	Maximum
Age (years)	22.57	21.50	3.45	19	32
Body mass (kg)	56.02	56.30	5.99	45.80	69.00
Height (m)	1.62	1.62	0.07	1.48	1.75
Body fat (%)	19.40	19.50	4.88	12.70	29.70
BMI (kg/m)^2^	21.35	21.45	1.55	19.31	23.82

Forty-three metabolites were identified. Some of them are known to be involved in energy metabolism, such as pyruvate, lactate, and glucose, which may be related to glucose/glyconeogenesis. Citrate, pyruvate, and succinate, which may be linked to the TCA, were also detected. The ω-hydroxy acids can be related to fatty acid degradation.

**Figure 1:**
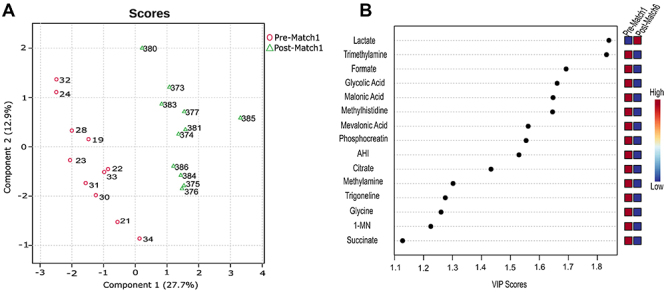
**A,** Plot of the scores from partial least squares-discriminant analysis (PLS-DA) at pre-match 1 and post-match 6. PLS-DA (Q2=0.62 and R2=0.78, permutation, n=1000, P=0.02). **B,** Variable Importance Prediction (VIP) score of component 1 was selected and metabolites with a VIP Score >1 are marked. AHI: Alpha-hydroxyisobutyrate; MN: Methylnicotinamide.

PLS-DA was used to assess the difference between the groups throughout the championships. The PLS-DA score graph shows the grouping comparing the first pre-match (1st match) and the last post-match (6th match) time-points (Figure 1A) and the VIP's metabolites more likely to be relevant to the grouping process, considering component 1 scores (Figure 1B). The models were validated by cross-validation and permutation, with Q2=0.62 and R2=0.78. The permutation test (n=1000) was statistically significant (P=0.02), indicating the models' reliability.

When component 1 was taken into account, the energy metabolites succinate, alpha-hydroxyisobutyrate (AHI), phosphocreatine, malonic and glycolic acids, citrate, 1-methylnicotinamide (1MN), and lactate (VIP>1) were responsible for the clusters observed throughout the championships (Figure 1B). Variations in the relative concentrations of metabolites at 6 pre- and post-matches are presented in the fold-change analyses (Supplementary Figure S1 and Supplementary Table S1). In total, 30 and 14 metabolites were considered significant before and after the matches, respectively, based on variations between mean relative concentrations before and after all matches (n=6) (Table 2).

**Table 2:** Significant differential metabolites between pre-match and post-match periods over the six official matches.

Differential metabolites	P value*	False discovery rate (FDR)
Pre-Match		
Trans-aconitate	1.7755E-7	7.6347E-6
Alpha-hydroxyisobutyrate	4.592E-7	9.8728E-6
1-Methylnicotinamide	8.4398E-7	1.2097E-5
Formate	3.2692E-6	3.5143E-5
Tartrate	6.7344E-6	5.7916E-5
Trigoneline	1.2573E-5	8.1837E-5
2-Hydroxyisovalerate	1.3322E-5	8.1837E-5
Leucine	1.5554E-5	8.3604E-5
Glucose	2.3468E-5	1.1212E-4
Uracil	4.2938E-5	1.8463E-4
TMAO	5.2034E-5	2.0341E-4
Trimethylamine	8.8339E-5	3.1655E-4
Tyrosine	1.0124E-4	3.3487E-4
Methyluric acid	3.3487E-4	4.7443E-4
Methylamine	3.2328E-4	9.2674E-4
Methylhestidine	4.4991E-4	0.0012
Mevalonic acid	8.0295E-4	0.0020
Glycolic acid	8.4748E-4	0.0020
Lactate	0.0013	0.0029
Creatinine	0.0015	0.0032
Isobutyrate	0.0016	0.0032
Methylguanidine	0.0016	0.0032
Hydroxyphenylacetic	0.0027	0.0051
Hippurate	0.0034	0.0061
Citrate	0.0036	0.0063
Succinate	0.0049	0.0081
Taurine	0.0058	0.0092
Phosphocreatine	0.0126	0.0194
Creatine	0.0167	0.0247
Malonic acid	0.0232	0.0333
Post-Match		
3-Hydroxyisovalerate	0.014	0.047
1-Methylnicotinamide	3.5251E-6	1.2248E-4
Alpha-hydroxyisobutyrate	9.5116E-6	1.3633E-4
Citrate	0.015	0.047
Glycolic acid	0.011	0.039
Hippurate	6.7954E-5	7.3051E-4
Methyluric acid	1.1909E-4	0.001
Methylhestidine	9.4689E-4	0.006
Malonic acid	0.007	0.034
Taurine	0.002	0.012
TMAO	0.008	0.03
Trigoneline	0.009	0.036
Trans-aconitate	5.6967E-6	1.2248E-4
Uracil	0.001	0.009

TMAO: Trimethylamine N-oxide; *Kruskal-Wallis test.

**Figure 2:**
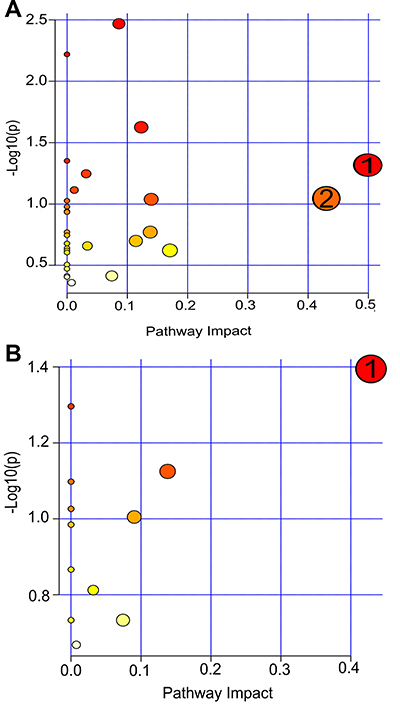
**A,** Metabolic pathways affected at the pre-match period over the six official games. 1: Biosynthesis of phenylalanine, tyrosine, and tryptophan (P=0.04); 2: tricarboxylic acid cycle (P=0.02). **B,** Metabolic pathways affected at the post-match period over the six official games. 1: Taurine and hypotaurine metabolism (P=0.04). Kruskal-Wallis test: P<0.05 corrected to the false discovery rate (FDR) and the analysis of the pathways considering impact scores >0.02 and -log(*p*) ≥1 and P value <0.05.

The differential metabolite analysis at pre-matches (Figure 2A) showed that tyrosine was responsible for the impact on phenylalanine, tyrosine, and tryptophan biosynthesis. The TCA cycle was also observed when pre-matches were analyzed, given its sequential occurrence. The hypotaurine and taurine metabolism pathways were the most impacted by the 14 differential metabolites in the post-matches period (Figure 2B).

## Discussion

Forty-three metabolites were identified in the urine of the 14 assessed female soccer players. Citrate, succinate, 1-methylnicotinamide, AHI, malonic and glycolic acids, lactate, phosphocreatine, and histone 3 protein (3HI) were the compounds originated from energy generating processes with high scores in VIP, impacting PLS-DA groupings. Based on the MetPA, the differential metabolites showed that phenylalanine, tyrosine, and tryptophan biosynthesis, as well as the TCA cycle were likely the affected energy production pathways, considering the pre-match moments. On the other hand, only taurine and hypotaurine metabolism were affected at the post-match period. Therefore, changes in the analyzed pathways seem to involve energy and amino acid metabolism.

Regarding 1-methylnicotinamide, AHI, malonic acids, and phosphocreatine [compounds associated with energy pathways and responsible for the clusters observed in the PLS-DA (VIP>1)], the possible explanation for the occurrence of these metabolites is the short time interval between matches, or even training in the days before official matches, since metabolite concentrations in urine can remain high for 1 to 3 days after exercise (19,20). A previous study showed that the degradation of phosphocreatine in urine after a single match is related to damage to muscle cells (15). However, we speculate that the breakdown of this metabolite may be due to the accumulated impact of each match on the body, as this metabolite is essential for energy production during sprints (20). In this sense, a previous study evaluating the effect of a game on saliva metabolites in female footballers described lactate as a marker of the degree of tissue hypoxia (12).

1-Methylnicotinamide resulting from NAD+ catalysis and activity seems to be related to enzymes associated with fatty acid oxidation (21-23). It was depleted after training in male soccer players during the winter season and remained high one day after training (19). Its detection may be due to its participation in the lipid oxidation process for energy supply, given that this metabolite is the result of NAD+ catalysis, which acts in the electron transport chain promoting the activity of enzymes associated with fatty acid oxidation (23). Malonic acid can be closely related to succinate dehydrogenase levels involved in the TCA cycle and accountable for converting succinate into fumarate (24). This metabolite can be related to the energy pathways mobilized during the matches.

The citrate, succinate, malonic, and glycolic acids observed in the VIP may be associated with the TCA cycle, and their detection may be due to fatigue accumulated over the matches, given that this pathway has been observed after induced fatigue in male soccer players (1). In our study, as mentioned before, there was a short interval between samples, as the championships were occurring simultaneously. Citrate and succinate are the main intermediates in the cycle and were detected in female players after a match (12), and citrate was also differential in the VIP in another study involving young male soccer players (1). Glycolic acid is an intermediate in glyoxylate metabolism (25), being associated with the energy pathway through the production of oxalacetate (26,27). On the other hand, 3HI is a ketone body that seems to act in energy provision (15).

The TCA cycle, phenylalanine, tyrosine, and tryptophan biosynthesis had an impact on energy production pathways at pre-match. The TCA cycle uses acetyl-Coa to produce energy through numerous reactions that can derive from macronutrients, carbohydrates, lipids, and proteins. In contrast to the present findings, the TCA cycle has been described in soccer players' urine metabolome after exercising sessions (1,12), indicating that citrate and succinate concentration disturbances and cycle components appear to be associated to fatigue in male soccer players (1). The impact of this pathway at pre-match can be related to the stabilization time of citrate and succinate levels in the body, which are the main TCA cycle intermediates identified in athletes. This was confirmed by a previous analysis involving healthy women's urine metabolome, which found that citrate levels increase at 24- and 72-h post-exercise (20). Games took place over a short period of time and, by speculation, one can say that this was a relevant fact to differentiate cycle intermediates at pre-match.

Phenylalanine, tyrosine, and tryptophan biosynthesis was influenced by metabolite tyrosine discrimination at pre-match, and it can be used to provide energy through its conversion into fumarate in the TCA cycle. A previous study pointed out that this pathway's regulation is associated with fatigue mitigation in mice (27). In addition to tyrosine, other amino acids play important roles in the muscle regeneration process (28), so its impact on phenylalanine, tyrosine, and tryptophan biosynthesis at pre-match may reflect the fatigue from previous matches, given the short time between them throughout the championships. Yet, previous studies have shown that tyrosine metabolism was affected in soccer players after fatigue induced in young athletes (1) and it has been one of the pathways involved in soccer players' adaptation to long-term training (8).

This pathway, along with other metabolic changes, could be useful to identify inefficient adaptations and to map the risk of muscle injuries in athletes (8). Tryptophan and phenylalanine are associated with oxidative stress reduction and protection against inflammation. Tryptophan acts in the kynurenine pathway, which is involved in regulating some organic functions, including inflammation and immune responses. Phenylalanine, in turn, is considered a tyrosine precursor, and can participate in both energy supply and in the synthesis of substances with anti-inflammatory functions (8,29-33).

Unlike the pre-match period, whose alterations were identified in the TCA cycle and in phenylalanine, tyrosine, and tryptophan biosynthesis, the taurine and hypotaurine metabolisms were affected at the post-match period. Taurine and hypotaurine have antioxidant properties (34), especially taurine, which protects muscles from damage by reactive oxygen species in soccer players (11) and acts as a muscle damage indicator during exhaustive exercising (35). Taurine seems to play a central role in this pathway, despite the antioxidant property. A previous study reported taurine's participation in energy processes, since it alters the use of energy substrates and decreases the use of glucose, in addition to favoring lipolysis during exercising sessions (36).

However, the impact of this pathway in the post-match period may be associated with muscle damage and fatigue due to the short time interval between sample collections, since taurine is an effective marker for assessing muscle damage (37). Another study suggested that the presence of taurine in urine samples after a match could be due to enhanced muscle damage in athletes (15). In this way, we believe that in the post-match moment there was an emphasis on an antioxidant action pathway to deal with reactive oxygen species, which increased over time during the championships.

**Figure 3:**
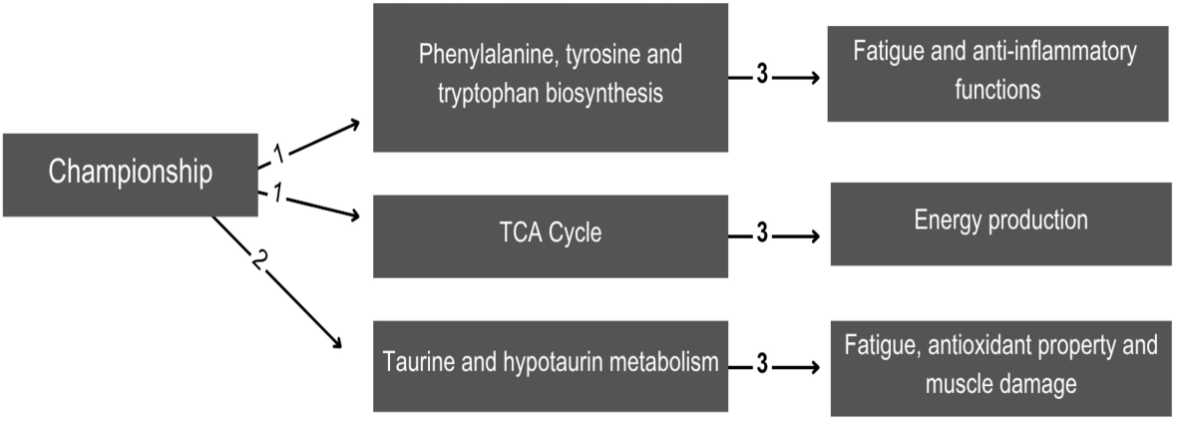
Metabolic impact in the context of the championships: 1) Metabolic pathways impacted in the pre-match period, 2) Metabolic pathways impacted in the post-match period, and 3) description of the pathways’ possible physiological action.

In summary, Figure 3 shows the main metabolites in pre- and post-match time-points over the championships and their possible physiological pathways.

Some limitations should be considered when interpreting the present results. The seasons' preparation periods could be included in the analysis for a better understanding of the matches' impact on metabolic pathways associated with energy production, although metabolomics is a robust technique capable of quantifying metabolites at specific times. Although the MetPA does not identify specific effects related to sports metabolism, to the best of our knowledge, it is the most efficient method for analyzing metabolic enrichment. In addition, each game has its own particularities, especially with regard to the effort required and the pathways mobilized in each match. Furthermore, information on food and water intake, sleep, and training load was not evaluated in this study. However, this is the first analysis describing urinary metabolites associated with the pathways mobilized in female football players during two simultaneous championships.

Responding to the hypothesis, metabolites linked to lipid and glycolytic metabolism were among the ones with higher VIP scores, together with metabolites related to protein catabolism. Future studies based on the present design must include a larger number of athletes, as well as preparation and end-of-season periods, so that metabolic information can be compared to other moments.

## Supplementary Materials

Supplementary Material Click to view [pdf].

## Data Availability

All data generated or analyzed during this study are included in this published article.
