# Supplementary material for: Energy production pathways of female soccer players during championships: a metabolomics approach
Source: Braz J Med Biol Res. 2026 Feb 16;59:e14589. doi: 10.1590/1414-431X2025e14589 (PMC12919752; doi:10.1590/1414-431X2025e14589)
Supplement: Supplementary Material [file 1414-431X-bjmbr-59-e14589-suppl.pdf]

**Figure S1.** Analyses of fold changes comparing pre- and post-matches over of the two championships: **A**, Match 1; **B**, Match 2; **C**, Match 3; **D**, Match 4; **E**, Match 5; **F**, Match 6. 3-AHI: 3-Alpha-hydroxyisobutyrate; MN: 1-Methylnicotinamide; TrA: Trans-aconitate; HPA: Hydroxyphenylacetic acid; 3-HI: 3-Hydroxyisovalerate. Fold-change analyses considered Log2 Fold Change = –1 or 1.

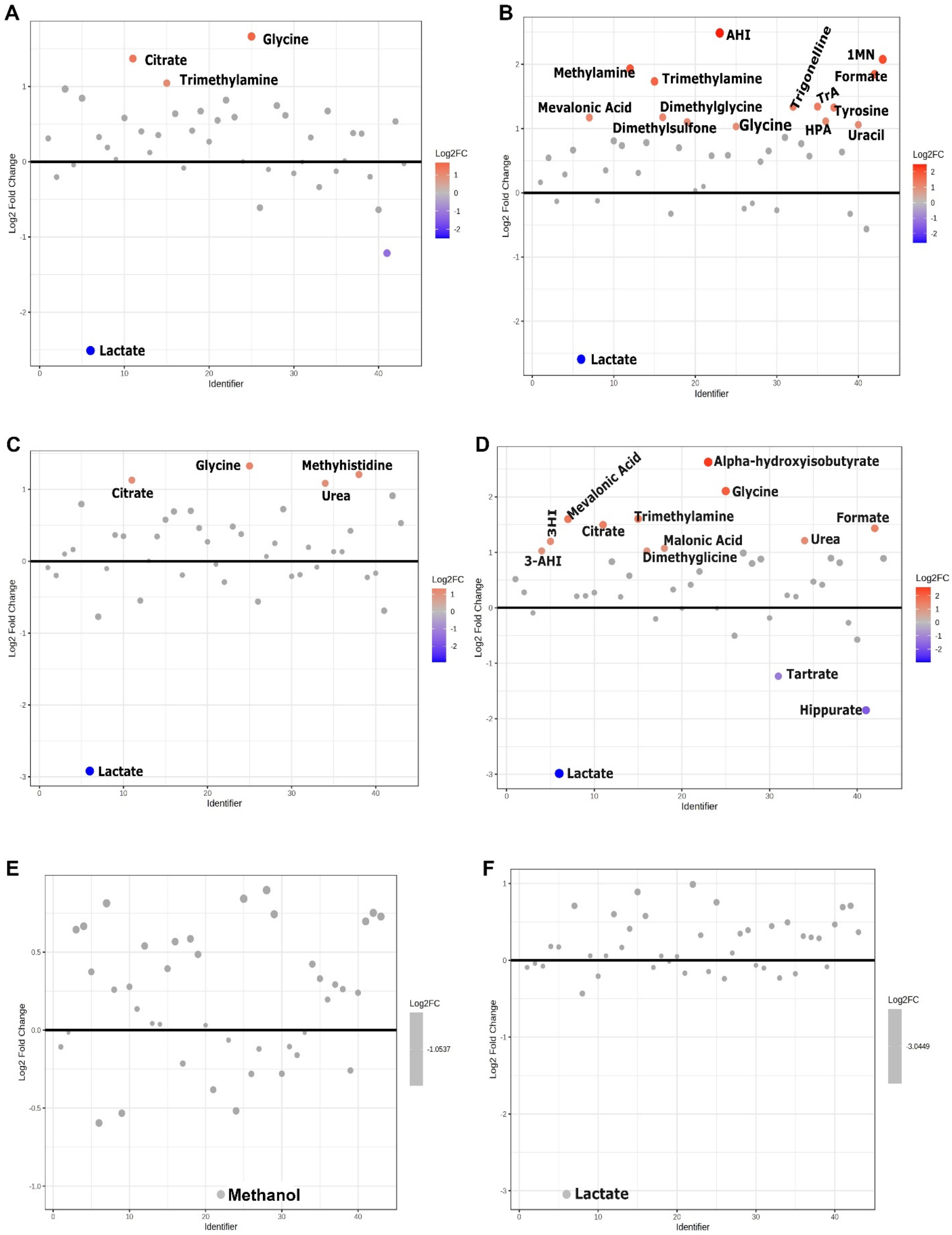

**Table S1.** Metabolites in the fold-change (FC) analysis comparing the pre-match and post-match moments in each match.

| Metabolites | Log2(FC) Match 1 | Log2(FC) Match 2 | Log2(FC) Match 3 | Log2(FC) Match 4 | Log2(FC) Match 5 | Log2(FC) Match 6 |
|---|---|---|---|---|---|---|
| 1-Methylnicotinamide | – | 2.07 | – | – | – | – |
| 3-Aminobutyrate | – | – | – | 1.02 | – | – |
| 3-Hydroxyisovalerate | – | – | – | 1.19 | – | – |
| Alpha-Hydroxyisobutyrate | – | 2.48 | – | 2.62 | – | – |
| Citrate | 1.36 | – | 1.12 | 1.49 | – | – |
| Dimethylglicine | – | 1.17 | – | 1.02 | – | – |
| Dimethylsulphone | – | 1.10 | – | – | – | – |
| Formate | – | 1.84 | – | 1.43 | – | – |
| Glycine | 1.66 | 1.03 | 1.32 | 2.10 | – | – |
| Hydroxyphenylacetic acid | – | 1.11 | – | – | – | – |
| Hypurate | –1.21 | – | – | –1.84 | – | – |
| Lactate | –2.50 | –2.59 | –2.91 | –2.98 | – | –3.04 |
| Malonic Acid | – | – | – | 1.07 | – | – |
| Methanol | – | – | – | – | –1.05 | – |
| Methylamine | – | 1.93 | – | – | – | – |
| Methylhistidine | – | – | 1.20 | – | – | – |
| Mevalonic Acid | – | 1.17 | – | 1.59 | – | – |
| Tartarate | – | – | – | –1.22 | – | – |
| Trans-aconitate | – | 1.34 | – | – | – | – |
| Trigonelline | – | 1.33 | – | – | – | – |
| Trimethylamine | 1.04 | 1.73 | – | 1.60 | – | – |
| Uracil | – | 1.05 | – | – | – | – |
| Urea | – | – | 1.03 | 1.21 | – | – |

– Metabolites without discrimination in the fold-change analysis for the match.
